# Patient public involvement (PPI) in health literacy research: Engagement of adults with literacy needs in the co‐creation of a hospital‐based health literacy plan

**DOI:** 10.1111/hex.13736

**Published:** 2023-02-28

**Authors:** Verna B. McKenna, Jane Sixsmith, Niki Byrne

**Affiliations:** ^1^ Health Promotion Research Centre University of Galway Galway Ireland; ^2^ Community Healthcare West & Galway University Hospitals Galway Ireland

**Keywords:** co‐creation, health literacy, health service responsiveness, knowledge translation, patient and public involvement, PPI

## Abstract

**Background:**

People with literacy needs can experience many challenges in accessing, understanding and using health services and health information. Such challenges can adversely impact patient‐provider interactions and ultimately, health outcomes. Healthcare providers need to be aware of health literacy (HL) to address the demands of healthcare systems, improve their interactions with communities and patients and promote patient engagement for improved health outcomes.

**Methods:**

This paper reports on a process of patient and public involvement (PPI) with participants in an adult literacy programme acting as PPI contributors to identify priority areas for a local hospital HL action plan and to develop a protocol for a PPI process with other groups. A qualitative community‐based participatory research study design informed by principles of PPI was undertaken, drawing on the tools of participatory and visual methods, open discussion and workshop format to facilitate a process of co‐creation. Three workshops with six PPI contributors took place to identify issues to be included in the hospital action plan. PPI contributors identified issues and grouped these into priority areas using discussion and ranking procedures.

**Results:**

Key areas prioritised for HL action by the PPI contributors were: verbal communication, emphasising the patient's right to understand, and improved understanding of medication use. These were incorporated into the action plan. The workshop format and process were deemed acceptable to the group and input on improvements will be incorporated into further work in this area.

**Conclusion:**

PPI acts as a lever in the knowledge translation process. Genuine engagement with service users can meaningfully contribute to relevant and sustainable changes to services as well as foster the empowerment of service users.

**Patient or Public Contribution:**

Members of the public with literacy needs actively participated in the co‐creation of a HL action plan for a local hospital and in the development of a protocol for a patient and public process for HL research.

## 1 INTRODUCTION

Health services are complex, and many individuals experience barriers to accessing and navigating these services. For individuals with established literacy needs, the potential for challenges is even greater.^1^, ^2^ It is increasingly acknowledged that healthcare organisations progress their responsiveness to such issues through system and practice improvements.^3^, ^4^, ^5^ Literacy involves understanding, evaluating, using and engaging with written text to participate in society, achieve one's goals and develop one's knowledge and potential.^6^ Health literacy (HL) is linked to literacy and represents the personal knowledge and competencies that accumulate through daily activities, social interactions and across generations.^7^, ^8^ Crucially, the World Health Organization (WHO) emphasise that such knowledge and competencies are mediated by both organisational structures and the availability of resources. These enable people to access, understand, appraise and use information and services in ways that promote and maintain good health and well‐being for themselves and those around them. In addition to the direct challenges posed by literacy needs, the associated stigma can also impair patient‐provider interactions and ultimately, health outcomes.^9^

In Ireland, 17.9%, or one in six, adults are at or below level 1 on a 5‐point level literacy scale, and approximately 40% have limited HL.^6^, ^10^ Healthcare providers need to be aware of HL to address the demands of healthcare systems, improve their interactions with communities and patients and promote patient engagement for improved health outcomes, behaviour change and treatment concordance.^11^, ^12^ The link between limited HL and health outcomes is well established. These include greater mortality, poorer overall health status, increased hospitalisations and emergency care use.^13^, ^14^, ^15^, ^16^, ^17^ In relation to limited HL and specific diseases research highlights, for example, associations with worse asthma severity, poorer diabetic control and obesity.^13^, ^15^, ^16^ Those with limited HL are more likely to be from disadvantaged backgrounds with lower levels of educational attainment, be older and from ethnic minority groups.^18^ Nevertheless, HL is found to be a stronger predictor of health outcomes than race/ethnicity, income, and education.^13^, ^19^, ^20^ Strengthening HL at a population level and making health services more accessible to those with low HL may be a useful strategy to reduce disparities and promote greater equity in health.^21^

Community‐based participatory research CBPR emphasises the importance of creating partnerships with the people for whom the research is ultimately meant to benefit.^22^ With a track record of approximately 25 years, CBPR has established its effectiveness in reducing inequities with its adherence to principles of co‐learning and health equity actions.^23^ The approach is closely aligned with ‘user‐led’ research.^24^ More recently the value of patient and public involvement (PPI) in health and social care research services is increasingly recognised.^25^, ^26^ PPI in research is defined as ‘research being carried out ‘with’ or ‘by’ members of the public, rather than ‘to’, ‘about’ or ‘for’ them’.^27^ PPI is based on the principle that healthcare should be person‐centred, involving patients and the public in the design, conduct and dissemination of research and improvement work.^28^ It involves the co‐production of health care through the use of active partnerships between patients and/or members of the public and researcher.^29^ Furthermore, PPI in health and social care research, policy and care delivery design is a mechanism that can promote the production of better HL.^30^ It builds on a growing interest in collaborative approaches to knowledge generation between knowledge users and researchers and that lead to ‘co‐created’ knowledge.^31^ PPI is underpinned by normative values associated with empowerment; change/action; accountability/transparency; rights and ethics.^32^ These values clearly align with the focus of HL on empowering individual citizens to demand rights and quality services and enabling engagement in collective health promotion action.^33^ People with limited literacy are less likely to have a voice in shaping health service planning, implementation and evaluation which can impact on the likelihood of their needs being met, potentially reinforcing inequalities. This work sought to develop and pilot a process for PPI in health services provision and aligned to the development of a HL action plan for a HLC at a local hospital. The HLC was established in 2015 to build a health‐literate responsive hospital in the context of quality improvement activities. It has developed and implemented a number of measures to address the HL needs of both patients and staff and also acts on the findings of the National Inpatient Experiences Surveys since 2017. Examples of activities undertaken by the HLC include the development, ratification, implementation and evaluation of a policy on written patient information; roll out of Plain English workshops for all staff; revision of outpatient and inpatient appointment letters; quality improvement of existing health information leaflets and booklets; and, hospital navigation evaluation using walking interviews with service users. While the committee included patient representation from its inception, the co‐creation of an HL plan with people with limited literacy has the potential to more meaningfully address their needs and those of all service users. This project sought to begin the process for ongoing PPI between PPI contributors, researchers and practitioners.

The purpose of this work was to engage people with literacy needs in the co‐creation of a hospital‐based HL plan. The objectives included: raising awareness of HL with recipients of adult literacy classes, introducing them to PPI and the Health Literacy Committee (HLC), exploring their experience in using health services, prioritising issues to include in the HL action plan, and identifying opportunities for further PPI contributions.

## 2 METHODS

### 2.1 Study design

A PPI approach was used within a CBPR study design. Specifically, this study drew on the tools of participatory and visual methods, open discussion and workshop format to facilitate a process of co‐creation.

### 2.2 Sample

Purposeful sampling was undertaken to identify persons attending adult literacy classes who could participate as PPI contributors. The Director of a local Adult Basic Education Service (ABES) provided expertise on the logistics of working with the PPI contributors. Initial dates coincided with increased Covid‐19 public health restrictions which resulted in delays as contributors were not available for online participation. The sample size was also impacted by Covid‐19 as the number of participants was restricted due to public health guidelines and room size requirements. Information sheets for potential contributors were drawn up which adhered to Plain English guidelines. These were distributed through the ABES by the Director. A total of six individuals (one male and five female) were available and willing to contribute. Feasible dates and times for the workshops were agreed upon with contributors through the ABES Director. In keeping with PPI guidelines contributors were provided with payment (in the form of a voucher) to offset the time given and basic travel expenses. All workshops took place at the ABES premises.^26^ Analysis was undertaken through engagement throughout the process. PPI contributors identified issues and worked together with the facilitator to group and prioritise these issues. The PPI contributors were involved in creating meaning from the data through the ranking process and group discussions.

### 2.3 Ethics

An Independent ethical review of the work was undertaken, and ethical approval was granted through the University of Galway Research Ethics Committee (2021.01.011) on 26 January, 2021.

### 2.4 Generation of ideas in the co‐creation process

Three participative workshop plans were developed by V. McK. and J. S. The workshops were all held at the ABES premises as this was most convenient and familiar for the contributors. Each workshop lasted two hours and took place during October and November 2021. Materials used included PowerPoint slides, flip‐charts, post‐it notes and visual images printed on A4 sheets. Participative activities were adapted, in part, from National Adult Literacy Agency (NALA) materials.^34^ All workshops ended with immediate session reflection for participants and facilitators. The purpose, methods used and outcomes of each workshop are detailed in Table 1. All workshops were facilitated by V. McK. and another researcher (E. V.), both experienced in working with marginalised groups.

**Table 1:** Overview of workshop purpose and activities.

	Purpose	Materials	Methods/activities
WS1	Introductions and rapport building. What is health literacy (HL) about? Your experiences Ground rules established: all opinions matter and no sharing of information outside the group	Power point Flip chart Post its A4 visual images	Visual images used to brainstorm on ‘what ways can you get health information?’ and ‘which do you prefer and why?’ Open discussions. Immediate session reflections. Brainstorm on the different health services you have used and your experiences in communicating with and understanding the services. What has helped or hindered? Explore usefulness of HL Communication techniques (Ask me 3 and Teach‐back method) Immediate session reflections.
WS2	Introducing the HL committee What it does How you can contribute	Power point Flip chart Post its A4 visual images	Reflections on what was identified at previous session. What could the HL committee do to address some of these issues? Immediate session reflections. Agreement on the issues that have come up. Ranking these in terms of importance Immediate session reflections.
W3	Prioritise what the HL committee needs to address/include in its action plan.	Power point Flip chart Post its A4 visual images	Discussed meaning and agreement reached on 3 issues to bring back to committee (co‐analysis). Discussed usefulness of the workshop plans for this process. Immediate session reflections

#### 2.4.1 Workshop 1

Purpose: This was aimed at introductions and building rapport between contributors and the facilitators. The contributors were introduced to the idea of ‘health literacy’. The open discussion then took place on their own experiences in using health information and health services. These discussions used activities adapted from NALA (2017) worksheets.^34^ Visual images of each idea were used, and participants were asked to add to these also. Ideas were recorded on flipchart sheets by facilitators (E. V./V. McK.) and transcribed by V. McK. following the workshop.

Activity 1: What ways do you get health information? Which do you prefer and why? Visual images provided of Internet, written information; demonstration; Smartphone; verbal.

Activity 2: Using your health services and communicating with providers. Identifying barriers and solutions. Facilitator (V. McK.) explained the ‘Ask me 3’ and Teach‐back methods as possible solutions and a discussion took place on how useful these might be.

### 2.5 Workshop 2

Purpose: Contributors were introduced to the work of the HLC and discussed processes to directly input into the committee's action plan. One of the facilitators (V. McK.) is also a participating member of this committee and so was able to address any questions on the work of the committee. Contributors emphasised the importance of having patients themselves help to design appropriate patient information materials, an area that has not been implemented to date by the committee, despite recognising the importance of such work. Contributors, with facilitators, discussed the issues recorded on the flipchart sheets and reached a consensus on ten key issues they believed to be important to improve how they could use the health services. These issues are listed below.

1. Understanding medication.
2. Provide help, for example, form filling.
3. Signage‐finding your way around.
4. Written‐patient information.
5. Verbal‐how staff communicate.
6. Treatment decision making.
7. Internet‐hospital site.
8. Promote the Teach‐back method/Ask me 3.
9. Videos on the website to explain more about a treatment.
10. Promote patient's right to understand.

**Figure 1:**
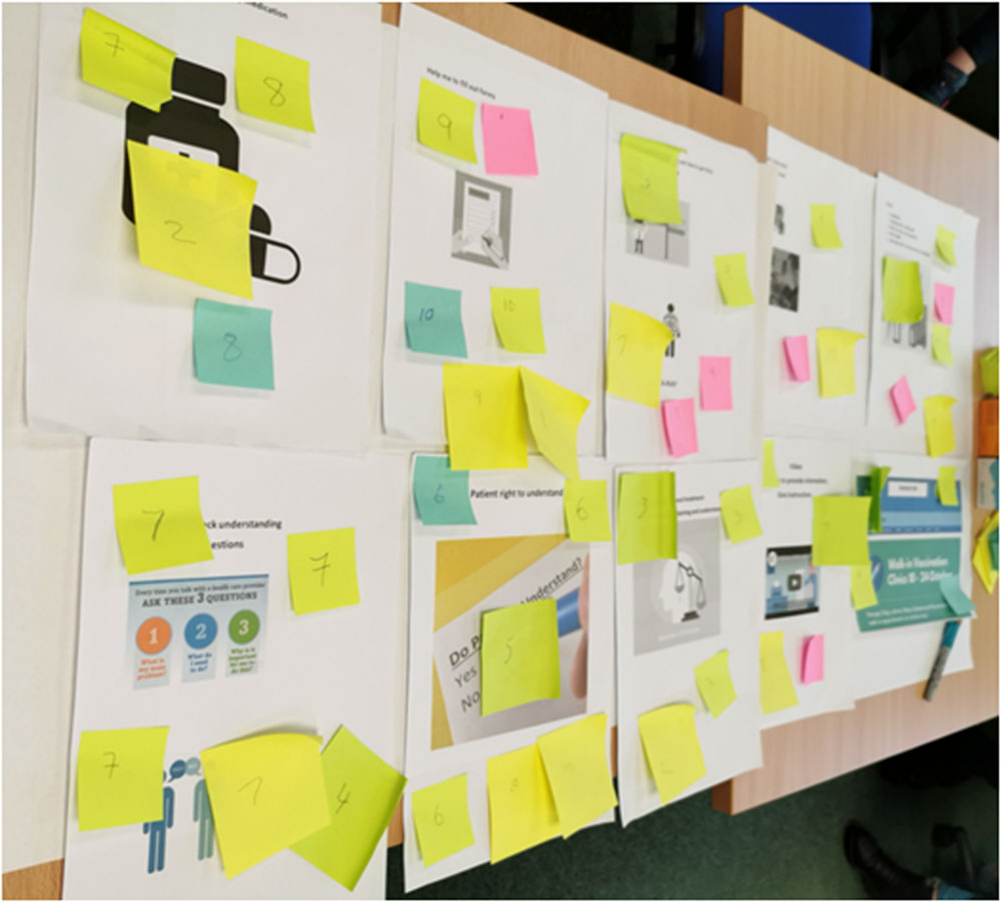
Ranking issues.

For workshop 2 each of these issues was represented by visual images on an A4 sheet and contributors were invited to rank each issue from 1 to 10 with ‘what do you think is the most important issue?’ being 1 and ‘what do you think is the least important issue?’ being 10. Contributors used a post‐it note to record a number from 1 to 10 and then attached this to the A4 image of the issue (Figure 1). The group were then able to see which issues they as a group prioritised. The session ended with a brief discussion on which issues were deemed to be most important. The facilitator (V. McK.) transcribed the issues, and rankings received to one list to be discussed at the next workshop.

### 2.6 Workshop 3

Results of the ranking process were discussed in more detail with three issues agreed on by the group of contributors based on the rankings received. These were:

1. Verbal communication.
2. Right to understand.
3. Help to fill out forms.

Group discussions then looked at the remaining seven issues and identified ways in which these could be incorporated into the three main issues. Discussion focussed on, for example, ‘what does verbal communication need to include?’ ‘What should my right to understand include?’ Agreement was reached on the following areas.

Verbal communication: This can be addressed by providers using the Teach‐back method.

Right to understand encompasses the use of ‘Ask me 3'; provision of accessible written information, use of videos and being able to understand how to use medicines.

Help to fill out forms encompasses help with navigation.

All of these decisions were brought to the HLC by V. McK. and incorporated into the hospital's 2022–2027 action plan for HL (see Table 2).

**Table 2:** Issues raised by contributors (in italics) mapped to action plan.

5‐year action plan for Health Literacy Committee	
Health Literacy Committee	Continue with established committee, with expanded staff and patient representative membership Identify Health Literacy Champions within the hospital Improve governance structures and build on reporting relationships Raise awareness of ‘Working with Patients with Limited Health Literacy' Massive Open Onine Course (MOOC) Continue to implement training plan on interpersonal skills and plain English writing.
Verbal communication	Increase/embed awareness of literacy needs among service users *Improve verbal communication with service‐users using explicit, evidence based and evidence informed health literacy communication techniques* *Implement rolling campaign focusing on improving verbal communication and emphasising the patient' sright to understand*.
Written patient information	Update the Policy on the Development of Written Patient Information (2017) and improve access and awareness of the policy Streamline governance and work‐streams for staff producing written patient information *Continue to improve quality of existing written patient information and ensure changes are maintained* *Implement process for user testing new materials with service users*.
National Inpatient Experience Survey and discharge planning	Highlight and address findings of the National Inpatient Experience Survey specific to the hospital Design and implement an intervention for the discharge planning process at the hospital, *with specific focus on understanding medication and treatment plans* Disseminate and implement patient discharge panning booklets during the admission process Evaluate any implemented actions.
Funding for health literacy activities	Identify funding opportunities and coordinate applications for funding Raise awareness with management and Allied Health Care Professionals (HCP) for funding opportunities.
Form filling and hospital volunteer team	*Advocate for assistance for service users with form filling across the hospital* Advocate to expand the volunteer role to build capacity for reducing Health Literacy demands on service users.
Physical environment improvements	Advocate for and assist with prioritising physical environmental improvements within the hospital maintenance plan, e.g., *navigation and way finding*.

### 2.7 Development of appropriate protocol/plan to involve other community‐based groups

Contributors engaged in the process of feedback at the end of each workshop session and focussed on what worked well? What did not work? And what could be changed next time? At workshop 3 contributors were asked to think about ‘how can we make these processes accessible to other groups in the community with literacy needs?’

Based on the feedback following each session the group considered the following process to be acceptable to work with other community groups:

1. Use the workshop delivery approach in familiar locations for participants so that they feel confident in attending and engaging in the group.
2. Raise awareness on HL with the community focussing on ‘what is HL and why is it relevant to us?’
3. Use the workshop activities to open up discussions. Use of visual images is really useful.
4. Do reflective feedback at the end of each session.
5. Prioritise important issues to be addressed using the ranking process.
6. Importance of having a tangible link to how contributions will be addressed. PPI contributors highlighted the benefit of having a clear linkage to bringing ideas to the table of the health care organisation. For example, it is important to know how the outputs from the workshops will be incorporated into the action plan of a working committee that can address the issues raised.
7. Provide a Plain English overview summary sheet of discussions to the contributors (this was compiled by V. McK. and sent to all contributors following the 3rd workshop).

## 3 DISCUSSION

Findings in this paper address both the organisational and community factors that affect HL.^35^ The development of an action plan contributes to reducing the complexity of the health system and improving how information and services are provided in a meaningful way. This paper draws on the principles of PPI to engage people with literacy needs in the co‐creation of a hospital‐based HL action plan and to develop a preliminary protocol for similar work with other groups based on the advice of the PPI contributors. It reports on the preliminary instigation of a PPI partnership for initial planning. Further, ongoing work will include PPI contributors working on implementation and evaluation activities. Embedding PPI at this early stage will support PPI sustainability in the long term for health literacy research with the HLC. This paper set out a pilot process for engagement with adult learners with literacy needs to contribute to the development of an action plan at a local hospital. The work was guided by a member of the hospital's HLC. The outcomes of the engagement process have served a number of important purposes. Firstly, the contribution of adults with literacy needs has provided a crucial element to the work of the hospital‐based HLC. Health literacy responsiveness has been defined as ‘the provision of services, programs and information in ways that promote equitable access and engagement, that meet the diverse HL needs and preferences of individuals, families and communities, and that support people to participate in decisions regarding their health and social wellbeing’.^36^ Embedding the input of users with literacy needs into the action plan ensures that the organisation is responding to identified needs and promoting ways to improve how services are delivered to maximise access, understanding and use of its services. This supports the WHO's definition of health responsiveness which emphasises the recognition and accommodation of ‘health literacy strengths, needs and preference to create enabling environments’.^37^ ^,p.x^ In addition, it supports the co‐design for health literacy‐responsive actions, one element of its action area to build health literacy‐responsive systems.^37^

Another outcome is the increased community visibility of the committee and the potential to increase PPI in its work going forward. It has also begun an important process of building capacity for HL research among marginalised groups. Agreement reached on the protocol provides a starting point for work with other groups. This will allow greater community reach for the HLC. For example, going forward a similar process can be used with other vulnerable and marginalised groups, such as asylum seekers. The process also raised awareness of HL and its importance for this group and thus benefits the PPI contributor.^38^ Contributors were very engaged with the concept and what it meant for how they couldengage with services in the future. For example, the meaning of HL was attributed to a right to ask questions, to be able to understand the purpose of different medications and to have the time to seek clarification from a healthcare provider regarding treatment plans. Issues identified as important by the PPI contributors are similar to domains of the Health Literacy Questionnaire (HLQ)^39^ which include feeling understood and supported by healthcare providers, appraisal of health information, navigating the healthcare system and ability to actively engage with healthcare providers. The outcomes of this process have addressed a gap that often exists between knowledge that is made available and its subsequent use. The direct input of PPI contributors acts as a lever for the implementation of such knowledge. The issues identified by the contributors have been incorporated into actionable tasks that the HLC will address within their 5‐year action plan. The contributors' input has emphasised, for example, a greater need to focus on communication in verbal interactions. The process has also led to opportunities to user‐test patient materials with this group in the future and thus increase the reach of the committee's work into the broader community. The outcomes from this engagement process have provided important patient input into what issues need to be addressed and how this can be done. Patient involvement is a crucial part of ‘a continuously learning health system’.^40^ and central to partnership working and shared decision‐making approaches.^41^ Genuine engagement with service users can enable us to make relevant and sustainable changes to services as well as foster empowerment of service users.

This paper reported on work at the beginning of a process that will contribute to quality and user experience improvements in the healthcare organisation and is aligned to the problem identification stage of innovation defined by Gabriel et al. (2017). The knowledge translation component moves the PPI contribution input to the second level of innovation, new ways of providing a service with scope for adoption and diffusion.^42^

### 3.1 Strengths

This work provides a real‐world example of knowledge translation relevant to health literacy. It has also developed a mechanism/protocol for working with vulnerable community groups to increase their knowledge of HL and what this means for health service access and use. PPI is still relatively new for HL research.^30^ The outcomes of this pilot have demonstrated a feasible approach for patients and the public to contribute to HL research in the future. The buy‐in of the adult literacy organisation's Director allowed us to address the obvious power imbalance between the researchers and the PPI contributors. The input from the Director at the beginning of the process to provide information on the process was extremely important in empowering the contributors to participate.

### 3.2 Limitations

Covid‐19 public health restrictions meant that the number of potential contributors had to be quite small to accommodate the room size. Nevertheless, 6 PPI contributors is an appropriate number in PPI research. A review of PPI in health literacy interventions documented a range of 3 to over 100 PPI representatives.^30^ Earlier plans to bring the PPI contributors and the HLC together to enhance engagement were not possible due to public health restrictions. PPI contributors favoured the idea of the ‘Ask me 3’ framework. The Ask Me 3® is a validated teaching tool created by HL experts to prompt patients and caregivers to ask specific questions regarding their treatment plan to enhance their understanding of their health conditions.^43^ While this approach is popular in HL practice, it is important to acknowledge that the evidence supporting its effectiveness is limited. However, its use is linked to increasing staff awareness of the importance of clear communication.^44^ Another study, linking the questions to videos, has also shown positive outcomes.^45^

### 3.3 Conclusion

In Ireland, the findings of the National Inpatient Experience Survey^46^ can provide leverage to embed HL responsiveness in healthcare organisations For example, the survey has consistently identified difficulties and dissatisfaction with the discharge planning process for patients. Meaningful improvement in this area should involve PPI contributors with literacy, health and social care needs to co‐produce the healthcare solution through involvement in aspects of research design.^30^

In this pilot work, PPI contributors acted as advisors. Further work will focus on involvement in other stages of the research process including prioritising areas for further HL research. The outcomes of the current study provide a useful map of how to do this through the involvement of potential service users. In addition, the contribution of people experiencing literacy challenges to the design of an action plan to address HL is an important part of ensuring that such action plans are effective, feasible and sustainable. Meaningful engagement of service users, particularly those who may be marginalised, can also empower people to assert their rights regarding health care and the right to understand. This in turn can act as a lever to mobilise healthcare organisations to become more responsive to the health literacy needs of the populations served.

## CONFLICT OF INTEREST STATEMENT

The authors declare no conflict of interest.

## Data Availability

Data sharing is not applicable to this article as no new data were created or analysed in this study.
